# Recent Progress of Development of Optogenetic Implantable Neural Probes

**DOI:** 10.3390/ijms18081751

**Published:** 2017-08-11

**Authors:** Hubin Zhao

**Affiliations:** Biomedical Optics Research Laboratory, University College London, London WC1E 6BT, UK; hubin.zhao@ucl.ac.uk; Tel.: +44-20-7679-0475

**Keywords:** optogenetics, optogenetic implants, optogenetic stimulation, neural probes, implantable device

## Abstract

As a cell type-specific neuromodulation method, optogenetic technique holds remarkable potential for the realisation of advanced neuroprostheses. By genetically expressing light-sensitive proteins such as channelrhodopsin-2 (ChR2) in cell membranes, targeted neurons could be controlled by light. This new neuromodulation technique could then be applied into extensive brain networks and be utilised to provide effective therapies for neurological disorders. However, the development of novel optogenetic implants is still a key challenge in the field. The major requirements include small device dimensions, suitable spatial resolution, high safety, and strong controllability. In this paper, I present a concise review of the significant progress that has been made towards achieving a miniaturised, multifunctional, intelligent optogenetic implant. I identify the key limitations of current technologies and discuss the possible opportunities for future development.

## 1. Introduction

Optogenetics is an emerging neuromodulation technique that can render neurons controllable by light. This technique combines genetic and optical methods to activate or inhibit specific neurons. Even though optogenetics is a comparatively fresh technique, using a light source to control neurons is not a brand-new idea. A very early application of optical neural stimulation was conducted in 1971 [1]. For the first time, blue light was utilised to accomplish targeted neural stimulation, and action potentials in Aplysia ganglia were successfully triggered. In 1999, Nobel laureate Francis Crick proposed the rudiment of optogenetics: a new optical method which could be used to trigger or silence specific types of neurons without any influence on other neuron populations [2]. Over the past two decades, numerous optical stimulation tools have been developed [3,4,5,6,7,8]. However, since these tools are mainly based on either the utilisation of exogenous cofactors or the expression of multiple proteins, they are difficult to be employed into in vivo applications [9]. In 2003, the expression of a single photosensitive protein, ChR2, was discovered [10]. It can be transgenically expressed into neurons, and then these neurons can be depolarised with blue light illumination [11]. After that, the new type of optogenetic technology was founded. Typically, blue light with ~470 nm wavelength is appropriate for ChR2 activation. This light-sensitive cation channel has been employed to realise precise spatial-temporal control of neuronal activities both in vitro [11,12] and in vivo [13,14,15,16,17]. While ChR2 is utilised to enable neurons with specific behaviour, there is also a need to develop a different tool which can silence neurons for a particular action. In 2007, a chloride pump, halorhodopsin (HaloR, also called NpHR for *Natronomas pharaonis* halorhodopsin), was revealed from the archaebacterium *Natronomas pharaonic* [18]. This pump allows neurons to be hyperpolarised by yellow light. NpHR typically requires yellow light source with peak wavelength of ~570 nm for activation. Then specific neuron behaviours could be inhibited with particular yellow light illuminations [18,19,20]. Since then, the field of optogenetics has significantly progressed.

As a cell type-specific neuromodulation method, optogenetics opens a new door for neuroprosthesis applications. In the past decade, neuroscientists have discovered numerous light-gated microbial opsins to activate, inhibit or bi-directionally manipulate targeted neuron populations [10,18,21,22]. In particular, the advance of ChR2 and NpHR makes relatively low-intensity optical neuromodulation possible [21,23]. They only require less than 1 mW/mm^2^ (ChR2) or 7 mW/mm^2^ (NpHR) light intensity to reach the threshold. Owing to this merit, many biological experiments (in-vitro or in vivo) have been performed to investigate complex brain circuitry and chronical brain illnesses, such as Parkinson's disease [24], epilepsy [25], blindness [26], etc.

Hence, there is an increasing need to construct novel optogenetic implants, using appropriate engineering approaches. These implants should be able to achieve precise light emission, and to reliably deliver light to targeted areas of brain tissue. Besides this, they should be capable of being applied in large brain circuitries for multi-site and multi-layer operations. They should also hold reasonable spatial resolution and suitable light intensity controllability. In particular, each stimulation site should be accessed and manipulated individually. In addition, it is desirable and meaningful to integrate neural recording electronics into these implants for closed-loop neuroprosthetics. Lastly, it would be advantageous if relevant safety and durability evaluation scheme can be created for these implantable devices.

In this paper, the state of the art of optogenetic implants is reviewed. The current achievements in optogenetic implant developments are highlighted, and their limitations are also identified. Based on different methods used for light delivery, optogenetic implants can be classified into two categories: wave-guided structure and direct µLED-on-optrode approach (optrode means optical probe). An optical waveguide is a physical component applied for light confinement and transmission [27]. A typical optical waveguide element is an optical fibre, and it has been commonly utilised for light coupling. In the µLED-on-optrode structure, µLEDs are selected as the light source. Rather than cooperating with waveguide elements, these µLEDs are directly bonded on the implant and inserted into brain tissue. In the following two sections, these two groups of designs are introduced and compared, and their strengths and weaknesses are also correspondingly pointed out.

## 2. Wave-Guided Structure

### 2.1. Laser-Based Optogenetic Implantable Probes

To date, different microfabrication techniques have been explored to develop advanced implantable optical stimulators. These optical implants aim to achieve local light delivery and multi-site activation with targeted luminance intensity and spatiotemporal resolution. One of the key factors for these implants is the light source. Optogenetic implants require suitable light sources, which could possess sufficient light intensity and can precisely deliver light into particular area of the brain. The laser, as a well-developed illuminant, has been broadly applied into optrode developments. It can provide strong coherent light with low divergence [28,29]. This guarantees high efficiency in light delivery. For laser-based optical implants, a wave-guiding structure is essential to be utilised for light steering [28,29,30]. In past few years, numerous wave-guided devices have been proposed to couple with laser sources for implantable light delivery.

**Figure 1:**
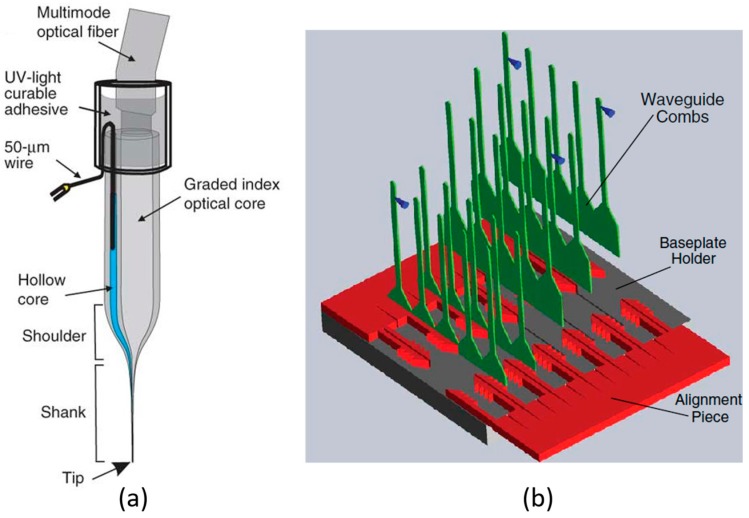
(**a**) Schematic diagram of the multimode optical fibre-coupled probe in [31]. A graded index optical core is coupled with the optical fibre to realise light delivery. A hollow core is used for the in situ observation of neural activities. The shaft tip is shaped to 10 µm diameter for single-neuron manipulation. This figure is reprinted with permission from ref. [31]. (**b**) Picture of the 3D waveguide optical array in [33]. This figure is reprinted with permission from ref. [33].

In 2011–2012, a glass fibre-coupled optical probe has been created by LeChasseur et al. and Dufour et al. for hybrid optical stimulation and electrical recording [31,32], as illustrated in Figure 1a. A single optical core is built into the probe for light emission, while a hollow core is utilised as the recording electrode. The optrode tip is formed with 10 µm diameter, and it provides single-cell resolution for light emission. A 100 nm aluminum coating is incorporated to minimise optical losses during light delivery. The maximum light intensity is around 10 mW/mm^2^. Shutters, dichroic mirrors, photomultiplier tube (PMT) detectors, and bandpass filters are incorporated to accomplish this optical-electrical microprobe system.

This optical probe holds several advantages. First of all, it possesses a very small probe tip. This facilitates the manipulation of single neuron or small populations of neurons. It also minimises the damage to brain issue caused by physical implantation. Besides, it achieves closed-loop integration in a single probe. Nonetheless, due to the limited number of stimulation sites (only one for each shank), it is challenging to employ this device in a large brain area for multi-site and multi-layer stimulation. Moreover, it requires several external instruments to complete the experiment set-up, which increases the total system dimensions. This may not be ideal for freely behaving animal experiments or clinical applications.

Instead, a wave-guiding optrode-MEA (microelectrode array) system has been developed by Jing et al. for concurrent optical stimulation and electrical recording [34]. This design is constructed based on a 6 × 6 Utah MEA; wherein one microelectrode is substituted by a fibre-coupled optical probe. The length of each microelectrode shank is 1 mm, and the pitch between every two microelectrodes is 400 µm. This optrode-MEA array achieves single site stimulation and 30-channel neural recording simultaneously, in millimetres cortical region.

This optrode-MEA array accomplishes large-region neuromodulation. Concurrent optical stimulation and electrical neural recording are obtained. However, it can only realise single-site stimulation within each 6 × 6 array, and multi-layer stimulation is also difficult to be achieved. In addition, the fabrication process of this device is comparatively complex, which might add difficulties for other researchers’ adoptions.

In order to expand stimulation sites into multi-layers, Zorzos et al. developed a 3-dimensional waveguide-based optogenetic array [33], as displayed in Figure 1b. This design consists of multiple linear probes in which individual microwaveguides are constructed in parallel, with variable length to accomplish multi-layer stimulation. The dimensions of output apertures in each probe are 9 µm × 30 µm. In this design, a laser source from Optoengine is utilised with 1.5 W power; then, an average maximum light intensity of 148 ± 56 mW/mm^2^ is generated. External optical coupling instrument is applied to realize light coupling.

Despite the device described above has achieved 3-D multi-layer and multi-site stimulation, this design is not without concerns. First, the power consumption is over high. It will limit battery lifetime and device operation period. Moreover, although this waveguide structure can accomplish multi sites, it is impossible to achieve individual operation for each site. More importantly, the used coupling instrument was bulky and cumbersome, thus it is difficult to apply this device for freely-moving animal experiments and/or clinical applications.

In 2017, Schwaerzle et al. proposed a silicon-based optical probe with integrated laser diode chips [35]. This optrode consists of two identical shanks, and each shank is with 8 mm length, 250 µm width and 50 µm thickness. The dimension of the probe base are 4 × 4 × 0.43 mm^3^. Two laser diodes are bonded at the probe base to generate two stimulation sites in each shank. Each shank includes two waveguides to guide the light from probe base to the emitting facets. An averaged light intensity of 96.9 mW/mm^2^ has been achieved. Four electrical recording sites are also constructed at each shank to accomplish simultaneous optical stimulation and electrical recording. In addition, based on this work, a dual-color probe has been developed in [36].

The work presented by Schwaerzle et al. prevents using bulky and cumbersome coupling instruments. Moreover, it achieves multi-site stimulation and in situ electrical recording. However, there is still some room to improve the spatial resolution of stimulation sites (currently only two on each shank). In addition, silicon is a relatively fragile material as probe substrate, the safety and long-term durability of the probe need to be carefully considered.

### 2.2. LED-Based Optogenetic Implantable Probes

Regarding the wave-guiding devices mentioned above, another concern is the laser light source. Even though laser systems can provide coherent light delivery with low divergence, there are still some areas to be improved, in terms of system miniaturisation, power efficiency, illumination stability, and operation warm-up period [28,29,30]. Additionally, laser systems usually require a dedicated optical interface for practical use [28,29]. This may add extra restrictions on the freedom of experimental subjects. Another type of light source used for optogenetic stimulation is the µLED. µLEDs could potentially provide more stable light emission. Some µLEDs could also hold faster light-switching speeds with lower power consumption [28,37]. Moreover, most µLEDs are compatible with conventional electronics for µLED driving and communication [28,29].

**Figure 2:**
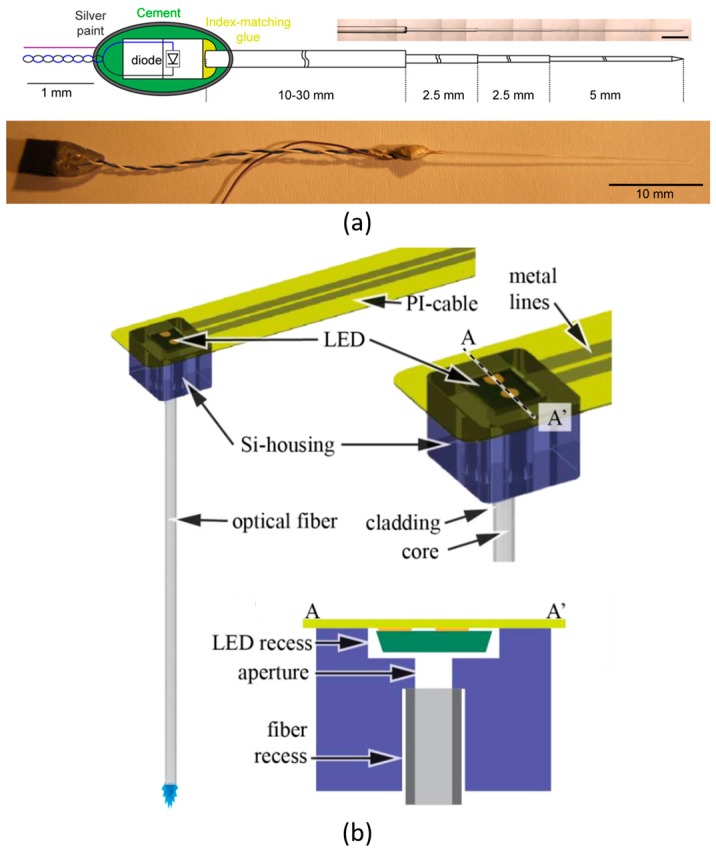
(**a**) Fibre-coupled multi-diode optical array in [38]. A single four-step fibre probe. The total length is 50 mm, and the last shank is 5 mm long with a 12° tip. This figure is reprinted with permission from ref. [38]. (**b**) Cartoon image of the fibre-coupled µLED optical probe [39]. This system mainly consists of a 270 × 220 × 50 μm^3^ LED chip, a flexible PI cable, a 550 × 500 × 380 μm^3^ Si housing, and a 5 mm-long optical fibre with 125 μm diameter. This figure is reprinted with permission from ref. [39].

Therefore, wave-guided µLED-based optical probes have drawn increasing attraction among neural-engineers. In 2012, an optical fibre-coupled multi-diode array has been developed by Stark et al. [38]. This array consists of six individual µLED-fibre coupled assemblies. The total length of the optical fibre is up to 50 mm, containing four stepped sections. The last section is 5 mm long with a 60–70 µm diameter. A cone-shape tip is constructed with ~12° at the end of the shank. A schematic diagram is shown in Figure 2a. This single fibre assembly is duplicated six times, to construct an optical array for multi-neuron operations.

This fibre-coupled optical array achieves multi-site stimulation by assembling six individual probes. Besides, it also realises adjustable-depth stimulation, as the four-step probe has high flexibility for stimulation depth. However, it does not achieve multi-site/multi-layer stimulation within a single probe. In addition, the system dimensions are relatively large. Both factors could be the bottlenecks for the use of this optical array for widespread applications.

Similarly, in 2014, another fibre-coupled µLED-based optical probe has been fabricated by Schwaerzle et al. [39], and the system overview is displayed in Figure 2b. The µLED is flip-chip bonded on a polyimide (PI) ribbon cable. The PI cable is bendable, ensuring the flexibility of the overall system. A silicon (Si) housing is implemented to adhere the µLED to the PI cable. A 5 mm-long optical fibre is fixed into a predefined recess of the Si housing to complete the system.

This wave-guiding µLED-based optrode can deliver light into deep tissue areas, with the incorporation of an optical fibre. It achieves 1.71 mW/mm^2^ light intensity with a 30 mA driving current and a 10% duty cycle. This meets the requirements of the ChR2 activation threshold (less than 1 mW/mm^2^). Additionally, it utilises relatively straightforward fabrication techniques, which could be adapted by other researchers. However, due to the utilisation of the bulky optical fibre, it would be challenging to multiplex this optrode for multi-site operations. Besides, although the light intensity reached the ChR2 activation threshold, it may be insufficient for higher-intensity stimulation. In addition, in order to achieve this 1.71 mW/mm^2^ light intensity, the consumed power is very high.

Based on the work presented in [39], in 2015, Schwaerzle et al. developed a 3 × 3 µLED fibre array for multi-site stimulation [40]. Although the design in [40] is more advanced, it has very similar limitations with the probe previously developed in [39].

Apart from all the devices described above, other fabrication technologies have also been explored, and diverse wave-guiding structures (both laser-coupled and LED-coupled) have been developed [41,42,43]. A performance summary of recently published wave-guided optogenetic implants is presented in Table 1. Even though these devices can meet the requirements of implantable optogenetic applications to some degree, they are still with some concerns. One limitation of some devices is that only one stimulation site could be constructed along the single probe shaft, which are not capable to be utilised for dispersed targets. In particular, due to comparatively bulky dimensions of some wave-guiding structures, the solitary light source is strenuous to be multiplexed for multi-site stimulations. For some other devices, although the single probe is duplicated into the array configuration to accomplish multi-site operations, the stimulation depth is still fixed, and multi-layer stimulation is not reachable within the single probe. This could potentially limit their applications in 3D brain network. Moreover, the light coupling efficiency of wave-guiding structures would be another limitation. Particularly for the µLED-fibre coupling efficiency, the maximum value is less than 10% [28,44]. This may increase the system power budget and reduce the light emission efficiency. In addition, more importantly, due to the incorporations of bulky wave-guiding structures and external optical communicators, these devices might be challenging to be applied into freely-moving animal experiments and/or clinical applications.

## 3. µLED-on-Optrode Structure

**Figure 3:**
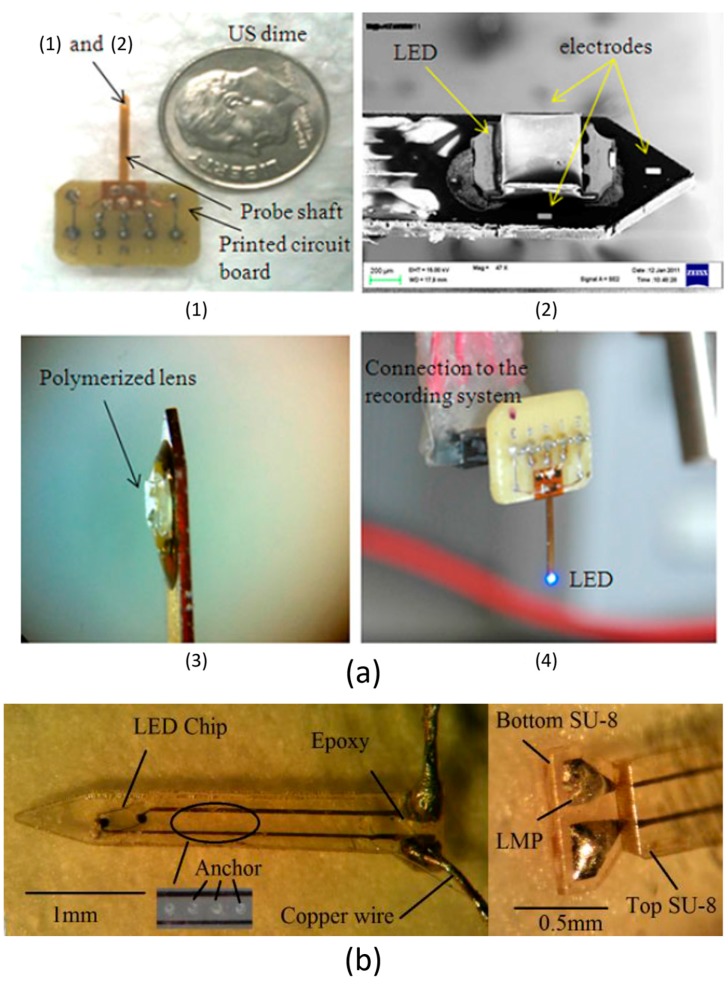

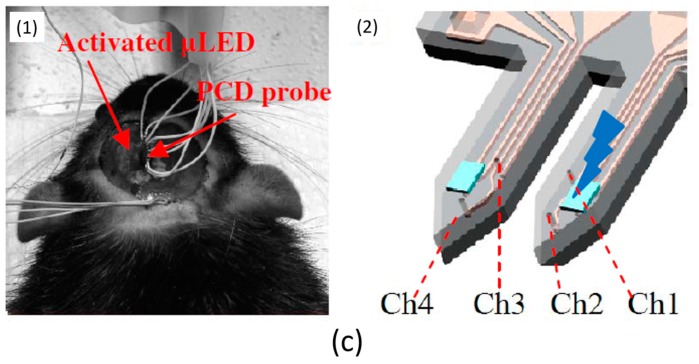
(**a**) Flexible polyimide-based µLED optrode in [45]. (1) A printed circuit board (PCB) is fabricated to assemble the optrode. (2) A scanning electron microscope (SEM) image of the optrode tip. The µLED site is bonded along with three recording sites. (3) A polymerised lens is covered on the µLED. (4) The overall system. µLED is turned on by a 2.9 V forward bias voltage. This figure is reprinted with permission from ref. [45]. (**b**) Samsung µLED mounted at the tip of the fabricated optrode in [46]. Several SU-8 anchors are created along the probe shaft, enhancing the bonding strength of two SU-8 layers. Cooper wire is bonded on the probe via the low melting point (LMP). Epoxy is adopted to further strengthen the bonding robustness. This figure is reprinted with permission from ref. [46]. (**c**) The PCD-based optical probe in [47,48]. (1) This PCD probe is inserted into an experimental rat for the in-vivo test. (2) A diagram of this two-shank probe. In each shank, the µLED is placed at the shaft tip, and two recording channels are positioned in the vicinity of stimulation site. This figure is reprinted with permission from ref. [48].

In addition to wave-guiding devices, another type of optogenetic implants relies on the µLED-on-Optrode structure. In this type of structure, µLEDs are straight bonded onto optical probes and distributed along the probe shaft. Then they are directly inserted into the targeted area of brain tissue. This strategy can provide higher light emission efficiency, and has the potential to realise multi-site/multi-layer stimulation within a single integral optrode. It could also free subjects from the movement restrictions caused by some bulky and cumbersome wave-guiding components. For instance, a flexible polyimide based integrated µLED optrode has been constructed by Cao et al. [45], as demonstrated in Figure 3a. In this optrode, one stimulation site is created at the probe tip, surrounded by three electrode recording sites. This optical-electrical hybrid design achieves optogenetic stimulation and simultaneous neural recording within a single probe. The whole shaft length is 12 mm, and the total width is 900 µm. An off-the-shelf µLED is utilised as the light emitter, with dimensions of 1000 × 200 × 600 μm^3^. The µLED drive voltage is 2.9 V, and the drive current is 5 mA. The light intensity of the µLED is fixed at 0.7 mW/mm^2^.

This integrated µLED optrode accomplishes both optical stimulation and electrical recording. Compared to wave-guided optrodes, it is more convenient for closed-loop integration. Besides, it requires less power budget and held better compactness. Nevertheless, there are several considerations for this design. Firstly, the µLED is too bulky. This would be a bottleneck both for realising multiple stimulation sites and minimising optrode dimensions. Furthermore, heating is a key concern with the µLED-on-optrode structure, but this design does not take µLED thermal effects into account. This may potentially induce over-heating in brain tissue. Also, this design requires 14.5 mW power to achieve 0.7 mW/mm^2^ light intensity, and so that the power efficiency has some spaces for enhancement. Furthermore, the generated light intensity is difficult to activate the ChR2, whose activation threshold is ~1 mW/mm^2^. In addition, the optrode shaft is too long, being much longer than the average cortex thickness [49]. It may need to be shortened in the future. Moreover, the optrode width of 900 µm might damage tissue and cause neuro-inflammatory responses.

At the same period, a two-layer SU-8 based optrode has been proposed by Fan et al. [46], and the fabricated optical probe is displayed in Figure 3b. The total length of the probe is 4.2 mm, and the width is 0.86 mm. The µLED (form Samsung Inc., Seoul, Korea) has dimensions of 550 × 600 × 200 μm^3^. A single stimulation site is formed at the optrode tip. The working threshold of this µLED is 2.6 V, and typical forward bias voltages are 3.0 V, 3.2 V, and 3.4 V. Corresponding drive currents and power consumptions are 11.2 mA/33.6 mW, 22.6 mA/72.4 mW, and 38.2 mA/130 mW. The maximum voltage is 3.6 V, generating drive current more than 60 mA (power consumption > 216 mW). When the µLED is biased with 3.4 V, the light intensity is around 0.9 mW/mm^2^; when the bias voltage is increased to 3.6 V, the light intensity would be approximately equal to 0.95 mW/mm^2^. If limiting the bias voltage to 2.74 V and constraining total power to 7 mW, the local temperature increase is restricted to 0.5 °C.

The SU-8 has outstanding flexibility, and this could decrease the tissue damage due to implantation. Also, this type of material is comparatively easy to fabricate. However, this SU-8 optrode is still with some concerns. Firstly, similar to the optrode previously mentioned (Figure 3a), the bonded µLED is large, which is not suitable for multi-site stimulation. Secondly, the light intensity generated is not strong enough. Only when the µLED is biased with 3.6 V voltage, it could nearly meet the ChR2 activation threshold. Higher light intensity is therefore required. Moreover, the power consumption/efficiency may need to be enhanced. Given 3.4 V and 3.6 V drive voltages, power consumption levels are 130 mW and 216 mW respectively. This may not be ideal for long-term implantable applications. In addition, although the thermal increment is limited to 0.5 °C with an input voltage of 2.74 V, thermal effects might need to be carefully investigated when the µLED is biased with the input voltages higher than 3.0 V.

Based on this SU-8 optrode, an improved work has been conducted by Fan et al. in [47,48]. Instead of using SU-8, a polycrystalline diamond (PCD) substrate has been constructed to obtain an integrated optical-electrical probe, as shown in Figure 3c. This optrode design has two different versions: single-shank and two-shank. For the two shank version, there are one µLED site and two microelectrodes on each shank, achieving optical stimulation and electrical recording; there is one more recording site on the shank. The same µLED as above (Figure 3b) is utilised. The length of each shank is 5 mm, and the width is 0.9 mm. The total dimensions of both versions of probes are 7.38 mm × 6.5 mm × 0.25 mm. The typical applied voltages are same to the previous work in [46], with light intensities of 0.6 mW/mm^2^, 1 mW/mm^2^ and 1.5 mW/mm^2^. In particular, due to the excellent thermal conductivity of PCD (up to 1800 Wm^−1^K^−1^), this optrode demonstrates outstanding heat dissipation performance. When the optrode is under 100 ms stimulation with 1 Hz pulses, the thermal increment is consistently below 1 °C.

However, although the PCD-based probe exhibits excellent thermal dissipation performance, there are still several points need to be considered. First of all, the light intensity produced is not fully sufficient for ChR2 activation. Only with a bias voltage of 3.6 V the ChR2 threshold could be triggered. A powerful light-driving ability is thus needed. Besides, the 0.9 mm shaft width of the probe is still over-wide, which may be likely to result in tissue injury and subsequent infection. In addition, the µLED dimensions are still a limitation for multi-site stimulation and optrode miniaturisation.

**Figure 4:**
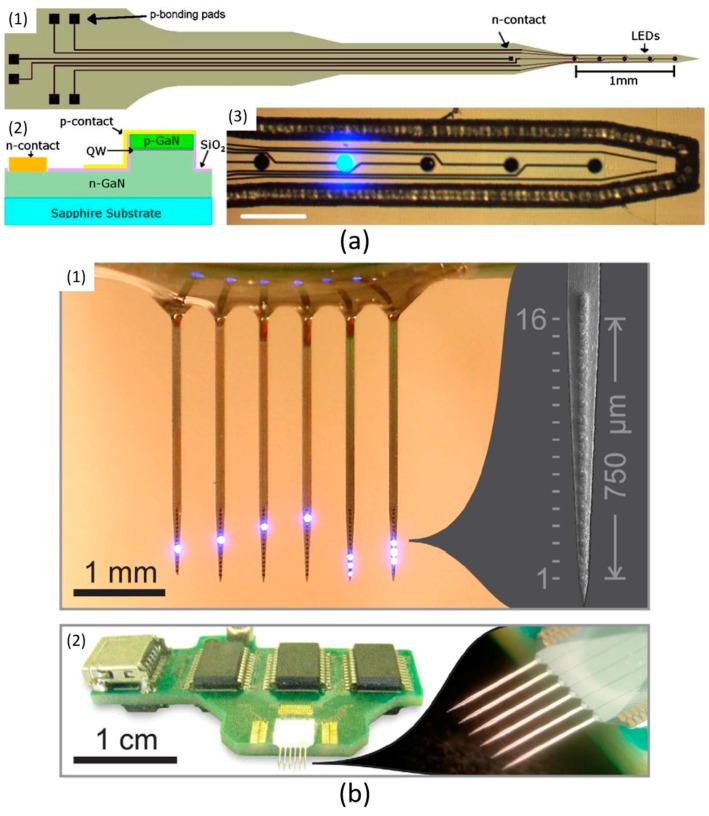
(**a**) Sapphire-based GaN µLED optrode in [50]. (1) System architecture of this optrode. Six bonding pads are placed at the head part to control corresponding µLEDs which are uniformly positioned along the optrode shaft. (2) The cross-section view of the fabrication process. (3) The optrode shaft including five µLEDs, one of them turned on with bright light. This figure is reprinted with permission from ref. [50]. (**b**) Silicon-based 6-shank GaN µLED optrode in [51]. (1) 16 stimulation sites are created on each shank, and they are uniformly distributed along the 750 µm shaft. Every µLED can be individually manipulated. (2) System diagram. The fabricated optrode is bonded on a dedicated control PCB. This figure is reprinted with permission from ref. [51].

Rather than employing commercially available µLEDs, a sapphire-based optical probe with custom-designed GaN µLEDs has been developed by McAlinden et al. [50], as shown in Figure 4a. The total length of this optrode is 7 mm, with a 1 mm-long shaft. The width of the optrode shaft is defined as 80 µm, to diminish the tissue damage caused by physical implantation. Five GaN µLEDs are evenly distributed along the shaft with 250 µm spacing. The diameter of each µLED is 40 µm. Six bonding pads are constructed on the optrode head. Five of these are anode pads with other a common cathode terminal. This configuration allows each µLED to be individually controlled. The maximum radiance intensity of this probe is 600 mW/mm^2^, and the maximum temperature increment is limited to ~1.5 °C.

This optrode accomplishes multi-site/multi-layer stimulation by applying the direct µLED-on-optrode structure. It obtains strong light intensity with a reasonable temperature increase. Nevertheless, there are still several considerations with this design. Firstly, the high material rigidity of sapphire may lead to tissue damage during or after insertion. Furthermore, the biocompatibility of both sapphire and GaN is a concern, so that further tissue infection might be caused. Besides this, due to the narrow shaft (80 µm), the future integration of neural recording electrodes would be problematic, which may prevent this device from being applied to closed-loop applications.

Based on the work illustrated in Figure 4a, an improved work has been completed in 2016 by Scharf et al. [51]. Instead of using sapphire, silicon is utilised to construct the substrate. An image of this silicon-based neural probe is shown in Figure 4b. This design consists of six optical shanks. Each shank includes sixteen GaN µLEDs along a 750 µm-long probe shaft. By incorporating ninety-six µLEDs, this 6-shank optrode provides high-density stimulations. It demonstrates outstanding spatial resolution, and is also able to generate sufficiently strong light intensity (~400 mW/mm^2^). A dedicated integrated circuit (IC) PCB is incorporated to control the probe, and each µLED can be individually addressed. Additionally, based on simulation results, with 150 mW/mm^2^ luminous intensity and 50 ms operation duration, the surface temperature increases by ~0.5 °C on average.

This brand-new optical probe has demonstrated several merits, such as high spatial resolution, strong radiance, reasonable thermal effects and the controllability/compatibility with conventional microelectronics. Even so, there are still some areas which may need to be improved. First of all, although this design demonstrates a reasonable thermal effects, these results are based only on simulation with a predefined light intensity, short operation duration and low repetition rate. It is desirable to analyse the thermal dissipation performance experimentally. In particular, even though in simulation only, the peak increment of temperature is around 4 °C. In actual use, this would be more severe if either the irradiance, working time and/or operation frequency are increased. Moreover, although the authors claims that recording electrodes could be potentially integrated into this system in the future, this might be challenging due to the narrow optrode tip and tight µLED spacing. Besides, for implantable applications, the PCB control board is relatively bulky and heavy. In addition, despite silicon being comparatively inert, biocompatibility with brain tissue may still need to be taken into account.

At the same period, Wu et al. proposed another type of high-resolution silicon-based optrode [52]. The optrode consists of four shanks, with three stimulation sites (µLED: 10 µm × 15 µm) and eight recording sites (11 µm × 13 µm). The shaft length is 5 mm, and shaft width is only 70 µm. With 13 mA input current, a light intensity of 353 mW/mm^2^ is produced. Moreover, each stimulation site can be individually controlled by external PCB control board. This silicon-based optrode achieves high-resolution, high-intensity, multi-layer stimulation. However, this design has similar limitations with the work presented in [51]: no experimental thermal analysis, bulky external PCB control board, and concerns about biocompatibility and long-term durability of silicon substrate.

**Figure 5:**
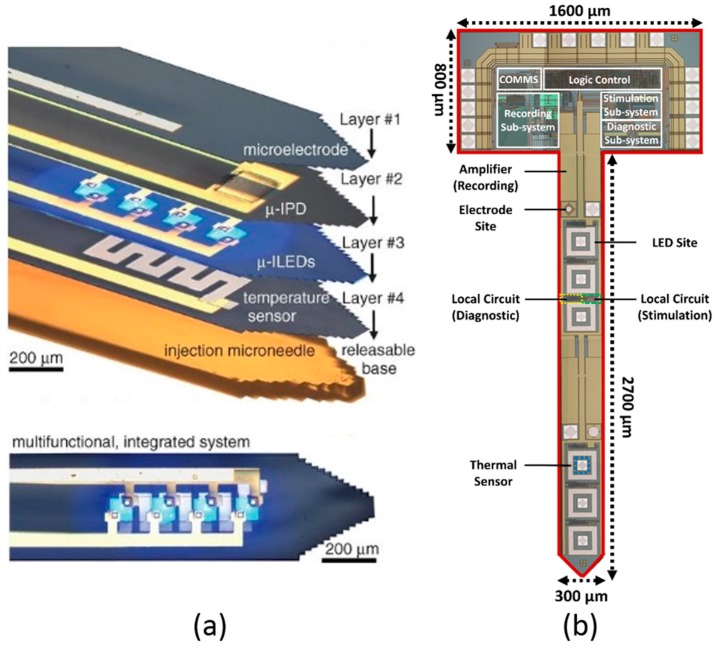
(**a**) Flexible electronics-based multifunctional implantable probe in [53]. Four different functional layers are incorporated along with a releasable base. The recording microelectrode is constructed on Layer 1; and then a micro-inorganic photodetector (µIPD) is utilised for photodetection at Layer 2; Layer 3 contains four micro-inorganic LEDs (µ-ILEDs) for optogenetic stimulation; and Layer 4 is dedicated to temperature sensing. This figure is reprinted with permission from ref. [53]. (**b**) CMOS-based active multifunctional optogenetic implant in [54]. Six stimulation sites are placed along the optrode shaft to be individually controlled. Self-diagnostic scheme is built into each site to enhance system safety and reliability. In situ recording electronics and thermal sensor are also accomplished at each site.

In contrast, a more advanced multifunctional injectable probe has been created based on flexible microelectronics by Kim et al. [53], as illustrated in Figure 5a. This design transforms the conventional solid optrode substrate into a flexible polymer base. Moreover, it integrates different material layers to achieve multiple functions, including electrical recording (Layer 1), optical detection (Layer 2), optical stimulation (Layer 3), and thermal sensing (Layer 4). In particular, a releasable base is built into this probe. During implantation, this injectable µneedle leads all of the functional layers into the targeted tissue region. After insertion, the µneedle is removed using dissolving fluid, and only the functional components are kept in the subject brain. The total thickness of all injected layers is only ~20 µm, which will greatly minimise any tissue damage induced by device insertion. The maximum light intensity is ~40 mW/mm^2^, which is sufficient for opsins activation. If the light intensity is limited to 17.7 mW/mm^2^, the temperature increase is restricted to 1 °C. Additionally, this device is compatible with wireless power transmitter, which could be highly beneficial for the freely-moving animal experiments.

This multifunctional implantable probe combines an optogenetic stimulator, recording electrode, photo detector, and temperature sensor in an integral device. It demonstrates excellent properties compared to the other devices reviewed above. Nonetheless, several concerns have been still identified and may need to be addressed in the future. One concern is the system thermal effect. The overall temperature increase can be limited to 1 °C, but this is on the condition of 17.7 mW/mm^2^ radiance. Given continuous 23.5 mW/mm^2^ light power, the temperature rise would reach 10 °C. Also, these thermal analysis results are obtained on the condition that the device is only implanted at a depth of 0.3 mm into the brain of the subject. The thermal increment would be higher with further insertion. Both the above situations may potentially cause thermal damage to the brain tissue. Another concern is its relatively low light intensity. This device is able to achieve a maximum light intensity of 40 mW/mm^2^. Although this value is much higher than the ChR2 activation threshold, it may not be sufficient for high-intensity deep-penetration applications. Moreover, under wireless control mode, only 7 mW/mm^2^ radiance is generated using 4.08 mW input electrical power. This indicates that the power efficiency still has some room for enhancement. Furthermore, the total length of the probe shaft is less than 1 mm, and this would become problematic for neural stimulation in deeper cortex areas. Besides, the construction of this multifunctional optrode relies on a costly and complicated custom-fabrication flow, which may result in restrictions for other researchers’ adoption. In addition, due to the system complexity, utilising a conventional microelectronic device to conduct logic control and two-way communication with the implant might be another challenge. Finally, operational degradation, such as electronic failure or mechanical breakage, might also need to be carefully considered.

Between 2014 and 2017, Zhao et al. utilised conventional commercially-available CMOS (Complementary Metal-Oxide-Semiconductor) process to develop a novel active intelligent optogenetic implant [54,55,56], as displayed in Figure 5b. A closed-loop version of the proposed optrode is constructed and fabricated using a 0.35-µm CMOS process. Six stimulation sites have been constructed and placed along the optrode shaft to realise multichannel neural stimulation. The maximum light intensity is 1256 mW/mm^2^, with a total power consumption of 6.04 mW when six LED sites are all on. An intensity magnitude control scheme is created which can adjust the LED drive current in different 256 levels. This provides an outstanding luminance controllability and ensures more accurate light delivery. This intensity magnitude control strategy could also minimise the over-heating hazard, confining the light power into a reasonable working range. Besides, the pulse width modulation mechanism is also implemented in this optrode design along with the intensity magnitude modulation, so as to attain satisfactory overall intensity modulations. More importantly, a self-diagnosis function is developed to monitor the optrode working status in real-time. If any abnormality occurs, either substrate breakage or µLED bonding erosion, it can be observed by the diagnostic sensing circuitry. Based on different scenarios, this will let user/clinical technician decide whether to switch to a backup µLED or to entirely turn off the implant. This self-diagnosis strategy profoundly enhances the functioning reliability and operational safety of the proposed optrode. In addition, an in situ resistor-based temperature sensor is developed to monitor the heating effect of the light emitters. These thermal sensors are placed within µLED pads, observing the real-time temperature on site. This thermal sensing design significantly heightens system robustness and, more importantly, makes sure of safe operation in brain tissue. Furthermore, electrical neural recording circuitry is incorporated into this implant. This can observe concurrent local neural signals while the targeted neuron populations are photosensitized by the optical stimulator. By employing the recording subsystem, a closed-loop neural interface is completed. This neural processing platform could be widely utilised for diverse neurological disorders, such as Parkinson’s disease and epilepsy.

The work described above is the first CMOS-based active optogenetic implant, but there are also some challenges within this device. First, this device requires relatively complicated post-processing to cut out the T-shape optrode. Moreover, CMOS technology is based on silicon substrate, but silicon is comparatively fragile, which may influence the longevity of the device. At last, although temperature sensors are incorporated into this system, the generated heat by LEDs might still be a concern.

Based on the literature survey conducted above, a performance summary of recently published µLED-on-optrode structure based optogenetic implants is presented in Table 2. Recent advances in µLED-on-optrode devices provide the feasibility to directly interact with deep brain tissues via optrodes. However, although these devices can achieve direct light delivery for optogenetic stimulation, their performances could still be enhanced from different aspects. Some devices could only obtain relatively weak light emission, which would be challenging for ChR2 (or other opsins) activations. Some devices can generate sufficient luminance, but they lack appropriate intensity modulation methods. Moreover, a very critical concern for implantable applications is safety. In particular, substrate integrity and operational degradation of light emitters are two dominant factors, which need to be taken into consideration. Besides, thermal dissipation is always a major concern for µLED-on-optrode structure. It is vital for both system stability and tissue health. Suitable thermal management is demanded, and temperature sensing circuitry is required to be integrated into the implant. Furthermore, electrical neural recording circuitry is desirable to be imported into the optogenetic implants, realising closed-loop integration. Some optical implants have incorporated recording electronics, but they use separate fabrication processes and/or control electronics from those used for the optical stimulators. This might increase the design and fabrication complexities, and cause difficulties for system integration. In addition, most of the current devices have been custom-fabricated. This might require comparatively huge financial and labour resources, and, more importantly, restrict their broader adoptions. Most importantly, except [54,55,56], all of these devices are controlled by external instruments/electronic systems. There are no active implantable electronics built inside to internally control the implants. All functions such as optical stimulation, electrical recording, and thermal sensing are performed via passive drive/control electronics. This may lead to a heavyweight for the overall system, and add obstacles for freely-moving animal experiments and clinical trials. Besides, their external control electronics are mostly not fully compatible with conventional biomedical microprocessors, which might limit their widespread applications.

## 4. Discussion

This paper has reviewed recently published work on optogenetic implants. Wave-guiding structure and µLED-on-optrode structure have both been investigated. The limitations of each reviewed device have also been identified. Following this, several observations are presented below.

### 4.1. Light Delivery Stability and Precision

For implantable optical stimulation, stable light delivery is demanded. Laser sources provide coherent light emission with low divergence, and µLEDs achieve more stable light delivery [28,29,30]. One concern of the wave-guiding structure is its low coupling efficiency with both lasers and LEDs [27]. Another concern is that, due to their bulky dimensions, the multiplexing of wave-guiding structures for multi-site stimulation is challenging. In recent years, vertical-cavity surface-emitting laser (VSCEL) demonstrates several merits over conventional lasers and µLEDs: low power consumption, narrow bandwidth, and high efficiency [57,58]. Blue-light VSCELs may be an optimal choice as emitters in optogenetic implants when they become commercially available in the future.

### 4.2. Optrode Dimensions

The optrode dimensions must be miniature. Several of the existing devices are comparatively large which may not be suitable for implantable animal experiments. The length of optrode shaft should be around 4 mm, achieving implant miniaturisation and matching the cortex thickness [49]. The shafts of some of the optical probes reviewed above are relatively short (around 1 mm or even shorter), which would make them unusable for deeper multi-layer stimulation. Optrode width and thickness (or diameter) should be within micrometre scale, minimising the potential tissue damage caused by device insertions. The head part of the implant should be of small dimensions and lightweight. The probe heads of several existing devices occupy large spaces, which may cause an extra burden for experimental subjects. Also, light emitters should be designed with minimal dimensions. This will help to increase luminance intensity, and facilitate multi-site stimulation, and also further minimise the implant.

### 4.3. Spatial Resolution and Temporal Resolution

The implant should be applicable to extensive brain network to allow the analysis of complex neural circuitry. In 2014, Shulyzki et al. [59] has developed a microchip based Utah-type probe to achieve 256 electrical recording sites along with 64 electrical stimulation sites. More interesting work have been demonstrated by Lopez et al. [60] and Angotzi et al. [61] to accomplish high-density neural probes. Likewise, optogenetic implants should hold appropriate spatial resolution (in the micrometre scale) for multi-site and multi-layer stimulation. Besides this, although the pulse frequency of stimulation operation is fairly low (typically less than 1 kHz), it is desirable to realise relatively high temporal resolution so as to improve the stimulation accuracy and efficiency.

### 4.4. Intensity Controllability

Light intensity controlling presently relies on manually changing the stimulation pulse width and/or shifting the driving current/voltage. It is desired and important to incorporate a more advanced control mechanism so as to achieve finer overall intensity modulation. This would be meaningful for optimising light/power efficiency, enhancing stimulation precision, improving operational safety, and regulating temperature increases [50,54,55]. Additionally, high-intensity emission is required, especially for deeper penetration applications.

### 4.5. Intelligent Implantable Electronics

To date, except [54,55,56], all of the existing optogenetic implants are passively controlled by different external controlling devices. This might increase the development complexity of the implantable system. Different implants require diverse dedicated controlling equipment, which might be cost-ineffective and labour-intensive. More importantly, these external controlling devices are not entirely compatible with those implants developed by other researchers. This would limit their broader applications. Besides, the relatively bulky dimensions and high power consumption of these controlling systems may further restrict the adoption of the implants in freely-moving animal experiments and/or clinical applications. Thus, there is an increasing need to construct an intelligent optogenetic implant for actual medical utilisations. Active controlling electronics should be built inside of the implant, which should be able to actively perform all required operations as a smart system. Standard communication protocol (such as Serial Peripheral Interface, SPI) should be embedded into the device, and moreover, this active implant should be compatible with general biomedical microcontrollers, so as to be easily adopted by the wider research communities and society.

### 4.6. Integrity and Degradation Evaluation

As the implant would be inserted into the brain, it will be difficult for clinical staffs/patients to observe its real-time working status. In particular, any breakage or component failure occurring in the implant may lead to high risks to patients’ health. Some preliminary analyses have been conducted in commercially available single-LED optrode [62]. However, in this review except [54,55,56], none of the studies published so far include a relevant evaluation strategy created within the implants. Thus, it would be advantageous to incorporate a dedicated sensing scheme for the evaluation of the system integrity and long-term durability.

### 4.7. Thermal Effect

Heat dissipation is of crucial importance in any optical implant, especially for the µLED-on-optrode structure. However, only a few existing devices take the thermal management into account. Detailed thermal analyses is essential to be conducted for proposed optrodes, and it is necessary to restrict temperature increases by a reasonable boundary (ideally within 2 °C [63,64,65]). Moreover, it is desirable for temperature sensors to be incorporated, which can monitor the system thermal effects in real-time. This will benefit both system stability and tissue health. 

### 4.8. Neural Recording Function

The use of a closed-loop neural interface is a growing trend in neuroprosthesis applications. The majority of current optogenetic implants are still conventional open-loop systems. For a new type of optogenetic implants, neural recording components should be built in. Local neural activities could then be observed to provide in situ feedback signals for stimulation operation. This could improve the system functioning performance, operational efficiency, and power efficiency.

However, when performing optical stimulation and electrical recording simultaneously, light-induced artefacts could be generated [66], and this usually caused by a photoelectric artefact called Becquerel effect [67]. This effect is challenging to be prevented and could potentially contaminate the electrical neural recording signals. One way is to utilise incoherent light source to reduce the Becquerel effect [67]. An alternative approach is to use transparent materials to construct recording electrodes to eliminate photoelectric artefact. Another method is to minimise the dimensions of recording electrodes to weaken the Becquerel effect [67]. However, either way cannot completely eliminate the photoelectric artefact. Other possible approaches should be continuously explored in the future.

To achieve the closed-loop integration, specific sophisticated closed-loop algorithm (such as spike classification, threshold detection) should be embedded into the control unit (e.g., PC, chest µcontroller) of the system. However, in the process of compiling this review, it is surprisingly common for this key characteristic to be omitted from most of reviewed literatures. The author thus suggests that reasonable description of algorithm implementation should be provided in the peer-reviewed closed-loop optogenetic implants in the future.

### 4.9. Fabrication Technology

Most existing optogenetic implants are custom-fabricated, which might be comparatively costly and labour-intensive. This may also create a barrier for widespread adoption and application. Thus, it is demanding to construct optogenetic implants using a commercially available fabrication process. One optimal choice is to utilise commercially available CMOS fabrication process. Lopez et al. [60], Angotzi et al. [61] and Shulyzki et al. [59] proposed active CMOS electronics for electrical recording/stimulation, and these works demonstrated the feasibility to utilise the probe shaft for active electronics. However, none of them is optoelectronic probes for the field of optogenetics. Then Zhao et al. [54,55,56] first proposed an active optrode for optogenetic stimulation with similar fabrication process with that of Lopez et al. [60], Angotzi et al. [61] and Shulyzki et al. [59]. To our knowledge, the work presented by Zhao et al. [54,55,56] is the first CMOS-based active optogenetic implant. This may open a new door for development of miniaturized optogenetic implant.

### 4.10. Power Consumption

Power consumption is also important for implantable applications, and it is critical for both battery operation and wireless power transmission. Most existing devices require strong current/high voltage, or their designs have not taken the power budget into consideration. It could be meaningful to optimise the system power consumption using low power design strategies such as power gating, energy harvesting, etc.

### 4.11. Substrate Materials

Variable materials have been utilised in these optogenetic implants as substrates. Polymer can demonstrate outstanding flexibility, but its high hygroscopicity may bias the long-term optical performance of the device. Diamond/sapphire materials have also been explored for probe constructions. These materials can provide finer thermal conductivity, but their strong mechanical rigidity may cause severe tissue damage and subsequent infections. Recently, silicon has also been utilised for substrate materials. Silicon can be potentially compatible with commercial CMOS process, however, its frangibility is a concern for long-term use. Thus, an “ideal” substrate materials should demonstrate good thermal conductivity, biocompatibility, robustness and flexibility, and should be easy for fabrication. This will require continuous efforts from materials and fabrication specialists to further push the technology forward.

## 5. Conclusions

To conclude, various fabrication technologies have been investigated and utilised for the development of optogenetic implants. Although these existing devices can meet the need of optogenetic stimulation to some extent, they have some areas for enhancement, in terms of illuminance stability, multi-site operation, intensity controllability, and miniaturisation. Moreover, it is desirable to incorporate different sensing and evaluation schemes into the implant. This could highly improve the overall system performance, with regard to safety, reliability, operation efficiency and functioning accuracy. Thermal sensing components, neural recording electronics, and integrity and functioning evaluation schemes should be built into the implant. More importantly, the targeted implant should be actively driven, acting as an intelligent standalone system. This intelligent implant should be fully compatible with conventional biomedical microprocessors, which can be widely unitised in the broader biomedical engineering field. In addition, the targeted implantable device is expected to be fabricated using a commercially available technology, which would be more convenient for other researchers to adopt. 

Optogenetics possesses great potential for treating neurological disorders. By photosensitising neurons via particular opsins, this method can be fully utilised for the investigation of complicated brain networks and neurological diseases. Potential applications are Parkinson’s disease, epilepsy, blindness, and other conditions. One of the key challenges is to construct appropriate optogenetic implants to deliver local light into the brain tissue of interest. This requires multidisciplinary effort from optical-electronics, biocompatible materials, precise fabrication, stable packaging, neuroscience and biology. The next five to ten years appears to be an exciting period for the development of new-generation optogenetic implants. It can be foreseen that multifunctional (optical stimulation, electrical recording, temperature sensing, etc.) intelligent device will be the trend of next-generation optogenetic implantable devices.

## Figures and Tables

**Table 1:** Performance summary of recently published wave-guiding structure based optogenetic implants.

Ref./Year	Light Source/Wavelength	Dimensions	No. of Sti Sites	Max Light Intensity	Max Power Consumption	Max Pulse Frequency	Electrical Recording	Fabrication Process	Substrate Material
[31]/2011	Laser/488 nm	Diameter: 200 µm; Shaft tip diameter: 10 µm	1	10 mW/mm^2^	-	-	Yes	Custom-Fabricated	-
[34]/2012	Laser/473 nm	Shaft length (L): 1 mm; Spacing: 400 µm	1	5 mW/mm^2^	-	40 kHz	Yes	Custom-Fabricated	-
[33]/2012	Laser/473 nm	Apertures: 9 µm × 30 µm	1 × 25	148 ± 56 mW/mm^2^	1500 mW	-	No	Custom-Fabricated	Silicon
[35]/2017	Laser/650 nm	Shaft L: 8 mm; W: 250 µm; laser diode dimensions: 300 × 300 × 100 µm^3^	2 × 2	96.9 mW/mm^2^	12.82 mW	100 kHz	Yes	Custom-Fabricated	Silicon
[41]/2011	Laser/473 nm, 593 nm	Shaft length: 7 mm; Width: 200 µm	1	-	21 mW (for blue light)	-	Yes	Custom-Fabricated	Polyimide
[42]/2013	Laser/473 nm	Shaft length: 5 mm; Width: 200 µm	1	9400 mW/mm^2^	50 mW	25 Hz	Yes	Custom-Fabricated	Silicon
[43]/2015	Laser/473 nm	Diameter: 150 µm	1	0.9 mW (~51 mW/mm^2^)	-	-	Yes	Custom-Fabricated	Silicon
[38]/2012	µLED/470, 589, 639 nm	Shaft L: 5 mm; Diameter: 60–70 µm (Blue) µLED dimensions: 1.6 × 0.6 mm^2^	1 × 6	40 mW/mm^2^ (blue light)	Current: 60 mA	-	No	Custom-Fabricated	-
[39]/2014	µLED/460 nm	Total length: 5 mm; Diameter: 125 µm; µLED dimensions: 270 × 220 × 50 μm^3^	1	1.71 mW/mm^2^	Current: 30 mA	-	No	Custom-Fabricated	Polyimide
[40]/2015	µLED/460 nm	Total length: 5 mm; Diameter: 125 µm; µLED dimensions: 270 × 220 × 50 μm^3^	1 × 9	1.28 mW/mm^2^	Current: 30 mA	-	No	Custom-Fabricated	Polyimide

**Table 2:** Performance summary of recently published µLED-on-optrode structure based optogenetic implants.

Ref./Year	Dimensions	No. of Sti Sites	Max Light Intensity	Max Power	Control Electronics	Integrity/Degradation Evaluation	Thermal Increment	Thermal Sensing	Electrical Recording	Substrate Material
[45]/2013	Shaft: 12 mm; Width(W): 900 µm; µLED: 1000 × 600 × 200 μm^3^	1	0.7 mW/mm^2^	Power: 14.5 mW	External instruments	No	-	No	Yes	Polyimide
[46]/2014	length(L): 4.2 mm; W: 0.86 mm; µLED: 550 × 600 × 200 μm^3^	1	0.95 mW/mm^2^	Power: >216 mW	-	No	0.5 °C increase with 7 mW power and 2.74 V input voltage	No	No	SU-8
[47,48]/2016	Shank L: 5 mm; W: 0.9 mm; µLED: 550 × 600 × 200 μm^3^	1	1.5 mW/mm^2^	Voltage: 3.6 V	External instruments	No	1 °C increase with 3.6 V input voltage	No	Yes	Polycrystalline Diamond
[50]/2013	L: 7 mm; Shaft L: 1 mm; W: 80 µm; μLED: 40 µm diameter	5	600 mW/mm^2^	-	External instruments	No	1.5 °C increase with 600 mW/mm^2^ and 200 ms pulse	No	No	Sapphire
[51]/2016	Total L: 3 mm; Shaft L: 750 µm; μLED: 25 µm diameter	16	400 mW/mm^2^	Current: 5 mA	External PCB control boards	No	0.5 °C increase with 150 mW/mm^2^ radiance and 50 ms pulse; Max: 4 °C	No	No	Silicon
[52]/2015	Shank L: 5 mm; W: 70 µm; μLED: 11 × 13 µm^2^	3 × 4	353 mW/mm^2^	Current: 5 mA	External PCB control boards	No	< 1.0 °C increase with 3.4 V voltage	No	Yes	Silicon
[53]/2013	Shaft L: 1 mm; W: ~400 µm; Thickness: ~20 µm; LED dimensions: 50 × 50 µm^2^	4	~40 mW/mm^2^	Power: 40 mW	External flexible/rigid control boards	No	1.0 °C with 17.7 mW/mm^2^ radiance and 10 ms pulse; Max: 10 °C	Yes	Yes	Platinum, Silicon, Polymer
[54,55,56]/2017	Shaft L: 4400 µm; W: 200 µm µLED dimensions: 20 µm × 20 µm^2^	6–18	1256 mW/mm^2^	Power: 6.04 mW	In-built active electronics	Yes	0.8 °C with 6 mW power	Yes	Yes	Silicon
